# Data on the agitation of a viscous Newtonian fluid by radial impellers in a cylindrical tank

**DOI:** 10.1016/j.dib.2017.10.035

**Published:** 2017-10-19

**Authors:** Houari Ameur, Youcef Kamla, Djamel Sahel

**Affiliations:** a Department of Technology, Institute of Science and Technology, University Center Ahmed Salhi of Naâma (Ctr Univ Naâma), P.B. 66, 45000, Algeria; b Faculty of Technology, Unisversity Hassiba Ben Bouali of Chlef, Algeria; c Department of Technical Sciences, University Amar Thilidji of Laghouat, Algeria

**Keywords:** Stirred tank, Rushton turbine, Paddle impeller, Power input, Flow patterns

## Abstract

In this paper, the data assembled concerning the agitation of a Newtonian fluid in a cylindrical vessel is disclosed. The stirred vessel is not provided with baffles and has a flat-bottom. The data presents some information on the characteristics of two impellers: a six-blade Rushton turbine and a six-blade paddle impeller. The flow patterns generated by both impellers are depicted and compared. Also, the power required when changing the impeller rotational speed is given. The data summarized here via three-dimensional calculations of velocities and viscous dissipation in the whole volume of the tank provides additional knowledge for the best choice of impellers for each industrial process.

**Specifications Table**

**Table:** 

Subject area	Chemical Engineering
More specific subject area	Fluid dynamics
Type of data	Figure, Table
How data was acquired	Based on three-dimensional calculations of velocities and viscous dissipation in the whole volume of the tank.
Data format	Analyzed
Experimental factors	The working fluid is the Glyerol solution.
Experimental features	The computer tool Ansys ICEM CFD (version 16.0) is used to create the geometry of the mixing system. Then, the computer code Ansys CFX (version 16.0) is employed to achieve computations. The equations of momentum and energy are solved by using the finite volume method. All calculations were performed in a computer machine having an Intel Core i7 CPU, 12.0 GB of RAM and a clock speed of 2.20 GHz.
Data source location	University Center of Naâma, Algeria
Data accessibility	Data is given in this paper.

**Value of the data**

• The data provide information on the flow structures and power consumption of radial impellers in mixing tanks.
• A comparison is made between two impellers: a Rushton turbine and a paddle impeller.
• The data presented here concern the case of Newtonian fluids.

## 1 Data

In this paper, we present the data obtained on the stirring of a viscous Newtonian fluid by two radial impellers operating in a cylindrical vessel. One Table and six Figures are included and which contain some information on the hydrodynamic and energy of these impellers.

## 2 Experimental design, materials and methods

### 2.1 Stirred system

**Fig. 1:**
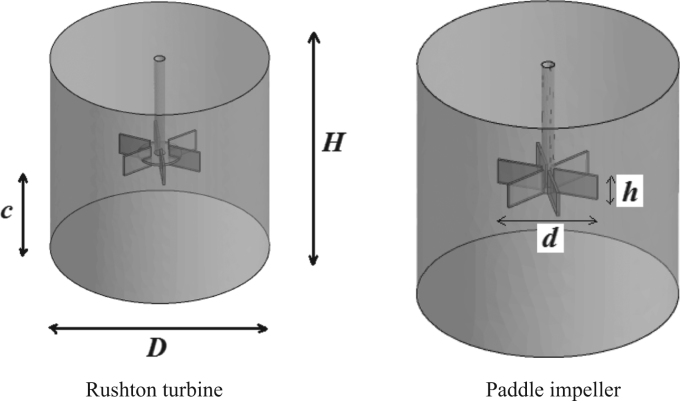
Stirred system.

The stirred system under investigation is presented on Fig. 1. It regards a cylindrical vessel having a flat bottom and not provided with baffles. Two impellers are explored, namely: a six-blade Rushton turbine (Fig. 1a) and a six-blade paddle impeller (Fig. 1b). Both impellers are placed at a concentric position and at the middle height of the vessel. The diameter (*d*_a_) of the impeller shaft is *d*_a_/*D*=0.06, and the disc diameter *d*_d_/*D*=0.2, with *D*=400 mm is the vessel diameter. The liquid level is equal to the vessel height (*H*). The required details of all geometrical parameters are given in Table 1.

**Table 1:** Geometrical parameters of the stirred system.

*D* [mm]	*H/D*	*h/D*	*d/D*	*c/d*	*d*_*a*_*/D*
400	1	0.1	0.5	0.5	0.06

### 2.2 Mathematical details

The Reynolds number (*Re*) for an agitated tank is defined as:

(1) Re=ρNd2μ

where *N* is the number of impeller revolutions (*ω*=2*πN*, *ω* is the angular velocity), *ρ* and *μ* are the density and dynamic viscosity of the working fluid (*μ*=1.5 Pa s), respectively. The Reynolds number is varying from 1 to 4×10^4^ and the standard *k-ε* model is used for modeling the turbulent flow.

The power number is calculated according to the following equation:

(2) $$N_{P}=\frac{P}{\rho N^{3}d^{5}}$$

where the power consumption (*P*) is calculated by integration of the viscous dissipation (*Q*_v_) in the whole vessel volume. The reader can find further details in our previous paper [1].

### 2.3 Data obtained

#### 2.3.1 Power consumption

**Fig. 2:**
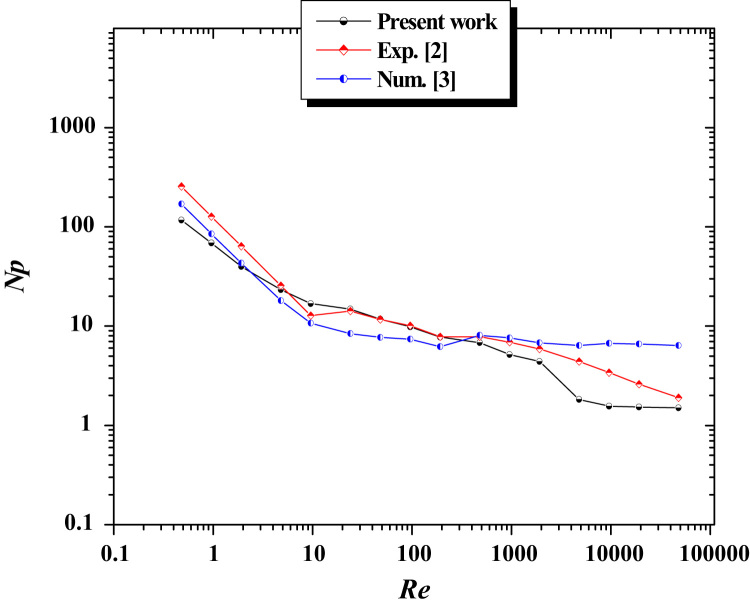
Power number for a paddle impeller.

In a logarithmic scale, values of the power number (*Np*) required by a paddle impeller are presented on Fig. 2 for different Reynolds numbers varying in a range covering the laminar, transitional and turbulent regimes. Our results and those obtained by Nagata [2] and Shekhar and Jayanti [3] are depicted and the same figure (Fig. 2) and all of these findings agree well. The increase of Reynolds number yields a great decrease in power number under laminar conditions. In the fully turbulent regime, *Np* becomes independent of impeller rotational speed.

#### 2.3.2 Flow fields

**Fig. 3:**
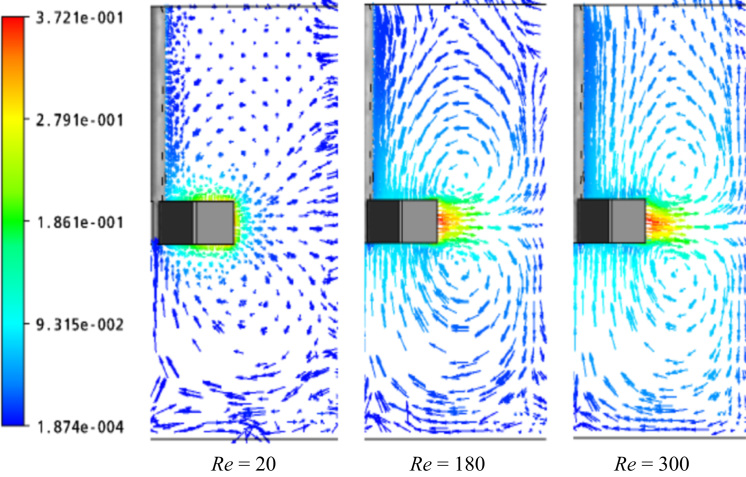
Flow patterns for a radial turbine at different Reynolds numbers.

The flows generated by a six-blade paddle impeller are depicted on a vertical plane passing through the impeller shaft (Fig. 3). We show here the effect of impeller rotational sped at low Reynolds number. Three values of *Re* are chosen, which are: *Re*=20, 180 and 300. These slices illustrate the radial jet of fluid particles impinging from the blade of impeller at a sufficient *Re* (*Re*=180 and 300). At low *Re* (*Re*=20), the flow is limited in the area swept by the impeller and the mixing is inefficient. However and with increased *Re*, the radial jet becomes more strong, giving thus an enhanced axial circulation.

**Fig. 4:**
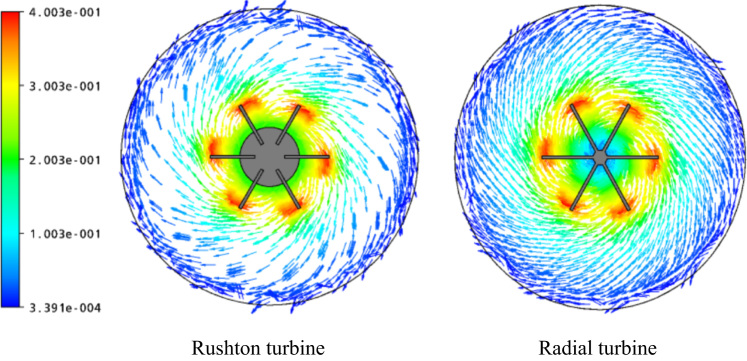
Velocity vectors for *Re*=3.1×10^4^, at R*=0.

**Fig. 5:**
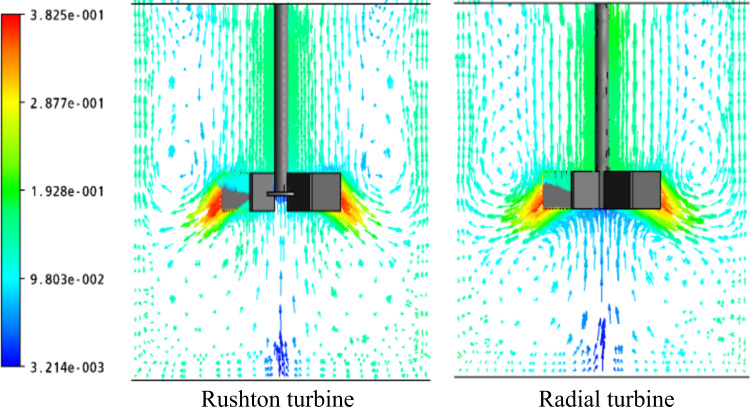
Velocity vectors for *Re*=2.9×10^4^, at R*=0.

Fig. 4, Fig. 5 provide a comparison between the Rushton turbine and a paddle impeller. For fully turbulent regime, the flow patterns are illustrated on horizontal and vertical planes passing through the impeller (Fig. 4, Fig. 5, respectively). The paddle impeller is characterized by its powerful radial jet than the Rushton turbine. However, the Rushton turbine gives a stronger tangential flow than the other impeller. This may affect the size of the well-mixed region, as reported in other studies [4], [5].

## 3 Data analysis

The data assembled is analyzed in Fig. 2, Fig. 3, Fig. 4, Fig. 5.

## References

[1] Ameur H. (2016). Mixing of shear thinning fluids in cylindrical tanks: effect of the impeller blade design and operating conditions. Int. J. Chem. React. Eng..

[2] Nagata S. (1975). Mixing- Principles and Applications.

[3] Shekhar S.M., Jayanti S. (2002). CFD study of power and mixing time for paddle mixing in unbaffled vessels. Chem. Eng. Res. Des..

[4] Ameur H., Kamla Y., Sahel D. (2016). CFD simulations of mixing characteristics of radial impellers in cylindrical reactors. Chem. Sel..

[5] Khapre A., Munshi B. (2016). Data on mixing of non-Newtonian fluids by a Rushton turbine in a cylindrical tank. Data Brief.

